# Returning to more finished genomes

**DOI:** 10.1016/j.gdata.2014.02.003

**Published:** 2014-03-01

**Authors:** Jonas Korlach

**Affiliations:** Pacific Biosciences, 1380 Willow Road, Menlo Park, CA 94025, United States

**Keywords:** DNA sequencing, De novo assembly, Reference genome, Consensus accuracy, Sequence read length, GC bias

## Abstract

Genomic data have become commonplace in most branches of the biological sciences and have fundamentally altered the way research is conducted. However, the predominance of short-read sequence data from second-generation sequencing technologies has commonly resulted in fragmented and partial genomic data characteristics. In this opinion, I will highlight how long, unbiased reads from single molecule, real-time (SMRT) sequencing now allow for a return to more contiguous and comprehensive views of genomes.

The generation of genomic data has revolutionized our ability to decipher the genetic blueprints of organisms, and thereby our understanding of the resulting biological phenomena and our means to biotechnologically and medically manipulate them. During the era of Sanger sequencing, a strong emphasis had been placed on the generation of comprehensive, *finished* genome information from de novo assemblies, despite the fact that this was laborious and expensive. While the advent of second-generation sequencing technologies provided significantly greater data throughput, their shorter read lengths and more pronounced sequence-context bias led to a shift towards resequencing applications, often limited to certain regions of those earlier reference genomes and focusing on single-base differences. The difficulties to produce finished genomes from short-read sequence data, even for smaller microbial genomes, resulted in a greater number of incomplete, highly fragmented, and often unannotated draft genomes [1].

The development of single molecule, real-time (SMRT) DNA sequencing has now made it possible to return to genomic data in the form of high-quality, finished genomes [2], [3]. This is because SMRT sequencing has excellent performance characteristics in all four areas that are relevant in the evaluation of sequencing technologies:

– *Accuracy*: for high-quality genomic data, the absence of systematic sequencing errors is imperative. Sequence errors in SMRT sequencing are distributed randomly and are read-length independent, resulting in consensus accuracies of > QV50 across genomes (less than one error in 100,000 bases), often exceeding what can be obtained with second-generation technologies [2], [3], [4].
– *Uniformity*: a prerequisite for comprehensive genomic data is the ability to sequence all the DNA that constitutes an organism's genome, irrespective of GC content of sequence complexity. SMRT sequencing has been demonstrated to exhibit the least degree of bias in sequencing data across different technologies [5], producing high-quality sequence even for extreme DNA sequence contexts [5], [6], [7], [8].
– *Contiguity*: the quality of genome assemblies is strongly dependent on the read lengths of the underlying sequence data [9]. The long, multi-kilobase reads in SMRT sequencing facilitate the direct resolution of repeats and other forms of structural variation to yield the correct genome organization [2], [3], [6], [10], [11], [12].
– *Originality*: because other sequencing technologies require DNA amplification, the vast majority of sequence data has been generated from DNA copies, not the original DNA that was extracted from the organism. In addition to the resulting amplification errors and bias, epigenetic DNA modifications are erased during amplification. SMRT sequencing does not require amplification, thereby eliminating such bias. SMRT sequencing also directly detects many types of DNA base modifications as part of the sequencing process (reviewed in [13]).

The scientific value resulting from these performance characteristics has been described in over 100 publications to date, spanning a wide range of biological application areas [14]. In several cases, the community has carried out direct comparisons of the quality of genomic data from different sequencing technologies, e.g. in the area of de novo assemblies of bacterial genomes [2], [3], [4], [7]. These publications signal a shift from fragmented and incomplete draft genomes from short-read sequence data, often represented by dozens to hundreds of contigs [3], to a new paradigm whereby fully finished, highly accurate microbial genomes can be obtained from SMRT sequencing data in an efficient, automated workflow, and several institutions have already implemented the routine generation of such high-quality genomes into their production workflows. The publications also highlight the importance for simultaneous fulfillment of the performance categories outlined above: for example, the GC-rich and repeat-rich genomes of *Streptomyces* strains have been very difficult to resolve with short-read technologies, resulting in over 450 contigs and over 10% of genome sequence missing due to large coverage gaps [7]. In contrast, the automated SMRT sequencing-based, near-finished assembly covered the entire 8.7 Mb genome in seven contigs, the largest of which contained > 90% of the genome [7]. It is also worth noting that genomic data characteristics strongly affect sequence depth requirements, resulting in marked differences between sequencing technologies. For example, in a study comparing assemblies of the *Potentilla micrantha* chloroplast genome, the authors noted that as little as 120 × SMRT sequencing coverage was required to generate a finished, 1-contig de novo assembly comprising the entire genome, while the corresponding short-read assembly was still fragmented and incomplete despite > 9000 × sequencing coverage, and was missing ~ 10% of the genome sequence [12].

While initially the new genome assembly methods utilizing the highly contiguous genomic data from SMRT sequencing were largely developed on microbial genomes, they are now being applied to larger genomes. Fig. 1 shows the de novo assembly for the yeast genome using the hierarchical genome assembly process (HGAP) developed for SMRT sequencing data [2], resulting in 30 contigs from the fully automated assembly workflow, relative to the 17 genomic elements (16 chromosomes plus mitochondrial DNA) present in the organism, i.e. each chromosome assembled into one or two contigs. With such high-quality assemblies, commonly used metrics to evaluate genome assemblies become less meaningful as they are more reflective of the organism's genome rather than the assembler's performance. For example, in this yeast assembly, the maximum contig length is 1.5 Mb because that is the longest genomic DNA element present in yeast (chromosome IV); it was assembled into a single contig.

A second example for more comprehensive genomic data from SMRT sequencing for larger genomes was demonstrated by an HGAP assembly of the *Arabidopsis* genome. Its comparison to results typically obtained with short-read technologies is shown in Table 1. The HGAP assembly contains the full genome (~ 12% was missing in the short-read assembly) with almost ten times fewer contigs, and almost 100-fold longer contigs on average. The longest contig spanned > 10% of the genome, and in several cases entire chromosome arms are represented as single contigs.

The performance characteristics of SMRT sequencing data are increasingly applied to the human genome, as well as other large and complex genomes [6], [8], [10], [11], [15], [16]. The lack of sequence context bias and the long read lengths have been employed to resolve regions that were previously difficult or even impossible to sequence by other methods, including attempts utilizing Sanger sequencing. For example, the gene encoding for MUC5AC, important for host-defense functions in the lung and other organs and implicated in cystic fibrosis and other diseases, contains a central large exon that had been intractable to sequencing due to its complex variable number tandem repeat (VNTR) structure, resulting in an ~ 50 kb gap in the human reference genome. By applying SMRT sequencing, a recent study demonstrated that this region could be resolved for the first time, and the high level of variation of this region between individuals was highlighted [6]. Similarly, in a paper entitled ‘Sequencing the unsequenceable’, 100%-GC DNA comprising the CGG trinucleotide repeat region in the FMR1 gene, responsible for fragile X syndrome, was shown to be amenable to SMRT sequencing [8]. Several groups have begun to apply SMRT sequencing over the entire human genome to leverage the long read lengths for the detection of various forms of structural variation, and to resolve regions which are difficult to access with short-read technologies due to their extreme DNA context or repeat content [15], [16]. Long SMRT sequencing reads have also been demonstrated to be valuable in transcriptome sequencing for resolving full-length transcripts and alternative splice isoforms [17], [18].

## Outlook

The high scientific value of finished genomes has been emphasized previously [19], as they constitute an important prerequisite for comparative and functional genomics, metabolic reconstructions, forensics, and many other fields. It is therefore important to establish standards for the quality of genomic data so that this level of genetic characterization can be reached more routinely. The performance characteristics of SMRT sequencing result in genomic data which more closely, comprehensively and contiguously reflect the organism's genetic and epigenetic constitution. New algorithms utilizing these data continue to be developed and optimized, e.g. HGAP, PacBioToCA, HBAR-DTK, PBJelly, Cerulean, and rDNATools to name just a few [20]. The resulting ability to generate high-quality, comprehensive genomic data in increasingly automated and cost-effective workflows is thereby anticipated to have a significant impact on improving our understanding of the genetic foundations of biology.

## Figures and Tables

**Fig. 1:**
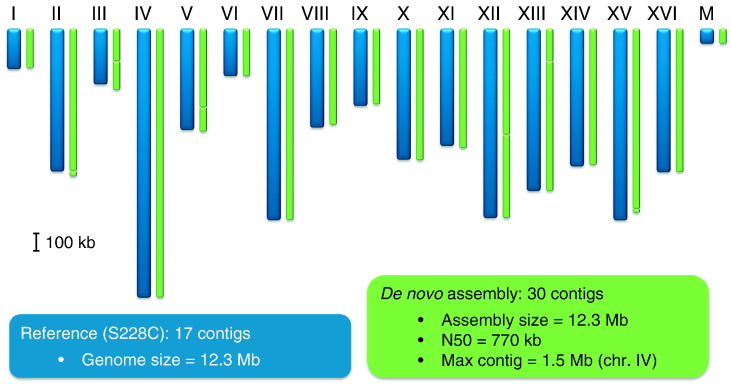
Yeast (*Saccharomyces cerevisiae*) de novo assembly (green) using SMRT sequencing and HGAP, and comparison to the reference genome (strain S228C, blue). Data available at http://pacbiodevnet.com/.

**Table 1:** *Arabidopsis thaliana* Ler-0 strain de novo assembly using SMRT sequencing data and HGAP, and comparison to a short-read assembly (Data available at http://pacbiodevnet.com/ and http://1001genomes.org/data/MPI/MPISchneeberger2011/releases/current/, respectively).

	PacBio assembly	Short-read assembly (2011)	Improvement
Assembly size (bp)	124,572,784	110,357,164	12%
# contigs	540	4662	8.6 ×
Contig N50 (bp)	6,190,353	66,600	90 ×
Max contig length (bp)	12,982,390	462,490	30 ×
